# Considerations and complications of mapping small RNA high-throughput data to transposable elements

**DOI:** 10.1186/s13100-017-0086-z

**Published:** 2017-02-15

**Authors:** Alexandros Bousios, Brandon S. Gaut, Nikos Darzentas

**Affiliations:** 1 School of Life Sciences, University of Sussex, Brighton, East Sussex BN1 9RH UK; 2 Department of Ecology and Evolutionary Biology, UC Irvine, Irvine, CA 92697 USA; 3 Central European Institute of Technology, Masaryk University, Brno, 62500 Czech Republic

**Keywords:** Transposable elements, Small RNAs, High-throughput sequencing, siRNAs, Genome mapping, Annotation, Bioinformatics, RNA-seq

## Abstract

**Background:**

High-throughput sequencing (HTS) has revolutionized the way in which epigenetic research is conducted. When coupled with fully-sequenced genomes, millions of small RNA (sRNA) reads are mapped to regions of interest and the results scrutinized for clues about epigenetic mechanisms. However, this approach requires careful consideration in regards to experimental design, especially when one investigates repetitive parts of genomes such as transposable elements (TEs), or when such genomes are large, as is often the case in plants.

**Results:**

Here, in an attempt to shed light on complications of mapping sRNAs to TEs, we focus on the 2,300 Mb maize genome, 85% of which is derived from TEs, and scrutinize methodological strategies that are commonly employed in TE studies. These include choices for the reference dataset, the normalization of multiply mapping sRNAs, and the selection among sRNA metrics. We further examine how these choices influence the relationship between sRNAs and the critical feature of TE age, and contrast their effect on low copy genomic regions and other popular HTS data.

**Conclusions:**

Based on our analyses, we share a series of take-home messages that may help with the design, implementation, and interpretation of high-throughput TE epigenetic studies specifically, but our conclusions may also apply to any work that involves analysis of HTS data.

**Electronic supplementary material:**

The online version of this article (doi:10.1186/s13100-017-0086-z) contains supplementary material, which is available to authorized users.

## Background

Across eukaryotes, epigenetic pathways contribute to diverse functions, including gene regulation and transposable element (TE) silencing [1]. Small RNAs (sRNAs) are a key component of these pathways. Numerous studies have investigated the biogenesis and functional roles of sRNAs, with most focusing on the molecular mechanisms that underlie these processes (for recent reviews see [2–4]). Some of these studies have utilized high-throughput sequencing (HTS) technologies, which generate vast numbers of sRNA reads. This capacity of HTS has facilitated the identification of novel sRNA classes, the quantification and comparison of sRNA expression profiles across tissues, and the discovery of genomic loci that map large volumes of sRNAs. These tasks have been supported by numerous computational tools, most of which have been tailored to study micro RNAs (miRNAs) [5–11], with fewer offering comprehensive identification, quantification and visual-based support for all sRNA types [12–17].

Even with these tools, significant challenges remain in the handling and interpretation of HTS sRNA data. An important one stems from the fact that some sRNAs map to unique locations (U_sRNAs) of a reference genome, while others align equally well to multiple locations (M_sRNAs). The handling of M_sRNAs is a major concern, as it impacts downstream analyses [15], and is as yet practically unresolved with different studies (reviewed in [18]) using different approaches and sRNA analysis tools. For example, the NiBLS method allows multiple mapping without any kind of normalization for the number of mapping locations [19], the SiLoCo tool of the UEA sRNA Toolkit weights each read by its repetitiveness in the genome [20], the segmentSeq package of Bioconductor allocates each M_sRNA only once to a predefined locus even if it maps to more than one place within this locus or indeed across the genome [13], Novoalign (www.novocraft.com) excludes M_sRNAs, and bowtie [21] and bwa [22] randomly place each M_sRNA to a single locus under their default settings. Finally, a recently updated version of ShortStack allocates M_sRNAs to single loci based on the densities of U_sRNAs [12, 18].

The importance of M_sRNAs and their handling may be dependent on the component of the genome under investigation; for instance, due to their repetitive nature, TEs are likely to map many M_sRNAs, which unavoidably complicates TE-related studies. This effect may be especially prominent in plants because of their large genomes (the average size of a diploid angiosperm is ~6,400 Mb) and the fact that most plant DNA has originated from TEs [23]. This point is exemplified by contrasting data from the unusually small genome of *Arabidopsis thaliana* (only 125 Mb of which ~24% is TE-derived) and the larger – but still small, relative to the angiosperm average – genome of maize (2,300 MB, ~85%). sRNA mapping studies have shown that <25% of *A. thaliana* TEs are mapped solely by M_sRNAs [24], but this increases to >72% for maize TEs [25]. Hence, careful consideration of M_sRNAs is crucial for understanding epigenetic processes in genomes like that of maize. The challenges of mapping sRNAs to TEs are exacerbated by the fact that accurate TE identification is a notoriously difficult task [26, 27]. To simplify the problem, previous studies have often used TE exemplars [28–30], each of which is a consensus of many TE sequences representing a single TE family or subfamily. The use of exemplars may be pragmatic, but it likely reduces the analysis resolution compared to examining whole populations of annotated TEs.

**Fig. 1:**
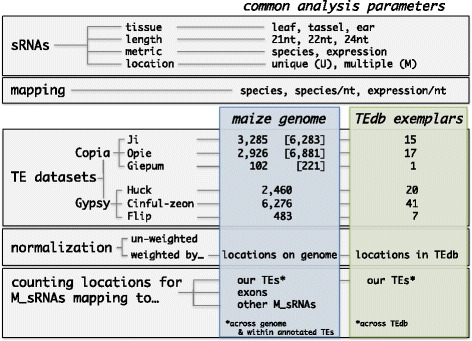
A matrix of the terms, data and analyses used in this study. The coloured boxes contain information specific for the maize genome (*blue*) or the TE exemplar database (*green*). The numbers in brackets for the *Copia* families represent their complete full-length populations retrieved from MASiVEdb

Here we attempt to address the complex, but understudied, issue of analyzing sRNAs in the context of TEs, because the impact of their treatment on analyses is presently unclear. To better assess different approaches, we focus on the maize genome and the most abundant *Copia* and *Gypsy* Long Terminal Repeat (LTR) retrotransposon families. We perform standard sRNA mapping using HTS data from three different tissues, but vary several features of the analyses, such as i) the reference dataset, which ranges from whole genome TE annotations to TE exemplars, ii) the treatment of M_sRNAs, which ranges from various normalization options to their complete exclusion, and iii) the sRNA metrics, i.e. consideration of distinct sequences or their abundances. Figure 1 depicts the methodological matrix of our work, along with many of the terms that we use throughout the study. We then comment on the effect of some of these choices on the relationship of mapping with other TE features such as TE age, with low copy regions of the maize genome, or when using HTS RNA-seq data. We conclude by sharing our insights as take-home messages to guide researchers in epigenetic analyses of TEs, especially in large and complex genomes.

## Methods

### TE reference datasets

We compiled two reference datasets for the *Copia* and *Gypsy* families in maize: annotated TE populations and TE exemplars.

#### Annotated TE populations

For *Copia* TEs, the Sirevirus families *Ji*, *Opie* and *Giepum* encompass the three most abundant families. *Ji* and *Opie* each constitute ~10% of the genome, and *Giepum* represents another ~1.2% [31, 32]. We used a strictly curated set of 3,285 *Ji*, 2,926 *Opie* and 102 *Giepum* full-length elements that were recently analyzed for their epigenetic patterns [25] (Fig. 1). For *Gypsy* TEs, we devised a pipeline to identify full-length elements of the three most abundant families, namely *Huck* (10.1% of the genome), *Cinful-zeon* (8.2%) and *Flip* (4.2%) [31]. We first retrieved the repeat annotation file from the maize TE consortium (‘ZmB73_5a_MTEC + LTR_repeats.gff’, ftp.gramene.org). This file, however, does not specify whether an annotated region represents full-length or fragmented TEs. Hence, we plotted the frequency distribution of the lengths of the annotated regions to identify peaks for each family that would correspond to the size of full-length elements as calculated by Baucom et al. [31] (Additional file 1: Figure S1A). This approach identified a single peak for *Huck* that nearly overlapped with the Baucom full-length average (13.4 kb), two peaks for *Cinful-zeon* that flanked the Baucom average (8.2 kb), and two peaks for *Flip* – one nearly overlapping with the Baucom average (14.8 kb) and one residing in close proximity (Additional file 1: Figure S1A). Based on these results, we selected regions between 13.3–14.1 kb for *Huck*, 7.1–7.5 kb and 9.2–9.7 kb for *Cinful-Zeon*, and 14.8–15.6 kb for *Flip* as candidates for full-length elements, retrieving 2,614, 6,965 and 607 sequences respectively. We then ran LTRharvest [33] with parameters *xdrop* 25, *mindistltr* 2000, *maxdistltr* 20000, *ins −*3, *del −*3, *similar* 50, *motif* TGCA, *motifmis* 1, *minlenltr* 100, and *maxlenltr* 5000 in order to identify the borders between the LTRs and the INT domain, and to also calculate the canonical LTR length of each family. Based on our approach, we selected LTR lengths between 1–1.8 kb for *Huck*, 450–750 nt for *Cinful-zeon*, and 4.1–4.5 kb for *Flip* (Additional file 1: Figure S1B), finally yielding 2,460, 6,276 and 483 full-length elements for each family respectively (Fig. 1).

The insertion age of each TE was calculated by first aligning the LTRs using MAFFT with default parameters [34] and then applying the LTR retrotransposon age formula with a substitution rate of 1.3 × 10–8 mutations per site per year [35].

#### TE exemplars

All maize TE exemplars were downloaded from maizetedb.org. The number of exemplars for the six *Copia* and *Gypsy* families ranged from one to 41 consensus sequences (Fig. 1). Note that we removed one *Ji* (RLC_ji_AC186528-1508) and two *Giepum* (RLC_giepum_AC197531-5634; RLC_giepum_AC211155-11010) exemplars from our analysis, based on evidence from [32] that they are not true representatives of these families.

#### Mapping sRNA and mRNA libraries

**Fig. 2:**
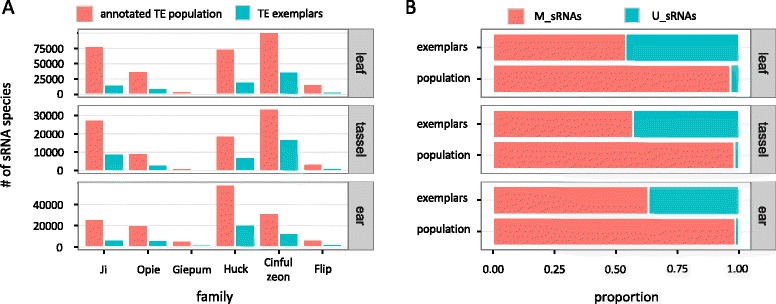
sRNA metrics on TE exemplars and annotated TE populations. **a** Total number of sRNA species that mapped to each family. **b** Proportion of U_sRNA and M_sRNA species for all families combined

We used published sRNA data from leaf (GSM1342517), tassel (GSM448857), and ear (GSM306487) tissue (Fig. 2), and mRNA data from three technical replicates (SRR531869, SRR531870, SRR531871) from leaf tissue. Adapters and low quality nucleotides were removed using Trimmomatic and the FASTX toolkit respectively, until every read had three or more consecutive nucleotides with a Phred quality score of >20 at the 3’-end. The libraries were filtered for miRNAs (www.mirbase.org), tRNAs (gtrnadb.ucsc.edu), and rRNAs and snoRNAs (rfam.sanger.ac.uk). sRNA reads of 21 nt, 22 nt and 24 nt length and mRNA reads longer than 25 nt were mapped to the maize B73 genome (RefGen_V2) and the maize TE database using bwa with zero mismatches (‘bwa aln –n 0’). Because bwa places multiply mapping reads randomly onto one mapping location under the default setting, we selected ‘bwa samse –n 100000000’ to ensure that all alignments were reported [22]. Following previous work [25], each distinct sRNA or mRNA sequence (of any length) was termed ‘species’, and the number of its reads was its ‘expression’. Each species was tagged as either uniquely mapped (U_sRNA; U_mRNA) or multiply mapped (M_sRNA; M_mRNAs) separately for the genome and the exemplar database (Fig. 1).

M_sRNAs and M_mRNAs were either normalized by their number of mapping locations or not normalized (Fig. 1), depending on the analysis. Finally, we calculated the total number of sRNA species that mapped to a TE ‘locus’ (i.e. the full-length sequence, LTRs or the internal (INT) domain), but also the number of sRNA species and sRNA expression (weighted or un-weighted) per nucleotide of each locus (Fig. 1). The per nucleotide measures allow comparisons of averages among TEs and also analysis along the length of the TE sequence.

## Results

### Reference datasets: TE exemplars vs. annotated TE populations

How do inferences vary as a function of the reference dataset? To investigate this, we compared sRNA mapping patterns between annotated populations and exemplars of six abundant families in maize. We focused on 21 nt, 22 nt and 24 nt sRNAs, because they are the sRNA lengths known to participate in the epigenetic silencing of TEs [36, 37].

#### sRNA mapping

We began by first examining the total number of sRNA species that mapped to each family. An initial observation was that there is a much lower number of sRNAs (3-fold decrease on average) that mapped to the exemplars compared to the annotated populations (Fig. 2a, Additional file 2: Table S1). For example, 90,503 sRNA species of the leaf library mapped to the exemplars of all six families combined, compared to 310,548 that mapped to the annotated elements.

#### U_sRNA and M_sRNA ratios

Previous research has suggested that U_sRNAs may exert a stronger effect on TE silencing compared to M_sRNAs, as evidenced by their more consistent correlation with DNA methylation [38], and with their association with lower levels of TE expression [24]. Accordingly, several studies have used only U_sRNAs as the basis for inference, derived either from mapping to genomes or to exemplars [29, 30, 39–41]. Our analysis showed that there is a massive difference in the U:M sRNA ratio as a function of the reference dataset: a much higher proportion of sRNAs map uniquely to exemplars (43% of all sRNAs for all libraries and families combined) compared to annotated TE populations (2.6%) (Fig. 2b, Additional file 2: Table S2). In fact, the vast majority of U_sRNAs that map to exemplars become M_sRNAs when mapped to the genome.

#### sRNA patterns along TE sequences

**Fig. 3:**
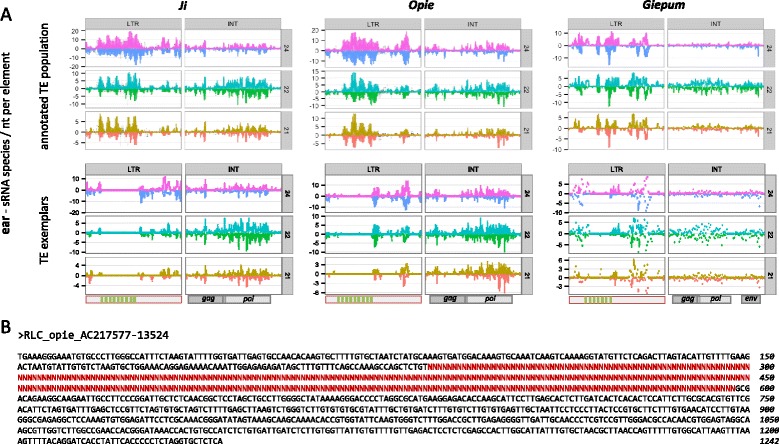
sRNA mapping along the sequences of *Ji*, *Opie* and *Giepum* exemplars and annotated populations. **a** Un-weighted sRNA data from ear tissue were mapped separately to the LTRs and the internal (INT) domain. Each region was first split in 100 equally sized windows, and mapping was calculated as the number of sRNA species per nucleotide of the sense (positive *y*-axis) and antisense (negative *y*-axis) strands, and visualized with a boxplot for each window. The position of the palindromes (LTRs) and the *gag, pol* and envelope (*env*) genes (INT domain) are shown at the bottom of each panel. **b** An example of the LTR sequence of an *Opie* exemplar with N nucleotides masking the unresolved palindrome-rich region

We next examined the mapping characteristics along the length of both exemplar and annotated TEs. We focused on the three *Copia* families, because of the preexisting annotation of their sequences, including information about complex palindrome motifs in the regulatory region of the LTRs that are sRNA mapping hotspots [25, 42]. We found that both datasets produced highly similar patterns, based on the ear sRNA library, with one intriguing exception: the exemplars were not mapped by sRNAs in the palindrome-rich regions (Fig. 3a). Closer investigation of the exemplar sequences revealed that they contain long runs of masked (N) nucleotides in these regions (Fig. 3b) of high sequence variability [25], even though they may be of special biological importance due to their elevated sRNA mapping and rapid evolution [25]. In fact, 74 exemplars from 37 families within maizetedb.org contain stretches of >100 N nucleotides (*Huck*, *Cinful-zeon* and *Flip* were not among them), making the occurrence of masked regions a fairly common feature of this dataset. The extent of this problem is not known for other plant species that have generated exemplar datasets such as foxtail millet [43] and strawberry [44]; yet, it now needs to be assessed, especially in the light of how helpful these datasets can be in combination with genomic, sRNA and RNA-seq HTS data in the analysis of the repetitive fraction of genomes [45, 46].

#### ‘Contamination’ of annotated TE populations

Our annotated TE dataset of the three *Copia* families is a curated subset of the complete population of maize Sireviruses available from MASiVEdb (bat.infspire.org/databases/masivedb/) [47], which comprises 6,283 *Ji*, 6,881 *Opie* and 221 *Giepum* full-length elements (Fig. 1) that have been identified as *bona fide* Sireviruses [48]. However, unlike our reference dataset, a number of these TEs harbor ‘contaminating’ insertions of other elements. Screening for foreign TE fragments within the two datasets using non-Sirevirus maize TE exemplars as queries (BLASTN, max *E*-value 1×10^−20^), we detected only two elements of the reference dataset with foreign TEs, compared to 1,158 elements of MASiVEdb that contained fragments (of 189 nt median length) from 451 non-Sirevirus families.

To examine how this might affect data interpretation, we compared the mapping characteristics of the reference dataset to those of the complete MASiVEdb population. The number of sRNA species that mapped to each TE family increased substantially for MASiVEdb. Collectively, 626,836 sRNAs from the three sRNA libraries mapped to the 13,385 TEs of MASiVEdb, but only a third (206,589) of that total mapped to our reference dataset (Additional file 1: Figure S2, Additional file 2: Table S1). Although it is difficult to assess the overall contribution of foreign TEs, given that even very small fragments may map several sRNAs, an indication may be provided by the level of sRNA ‘cross-talk’ within each dataset, that is the extent to which sRNAs map to multiple families. Our conjecture is that higher levels of cross-talk in MASiVEdb will reflect the presence of fragments of one family within elements of another family, thereby artificially increasing their pool of ‘common’ sRNAs. Our analysis showed that indeed this was the case. For example, of the 800,421 sRNA species of all libraries combined that mapped to *Ji* and *Opie* from MASiVEdb (Additional file 2: Table S1), 188,926 mapped to elements of both families. This means that the number of non-redundant sRNAs between *Ji* and *Opie* is 611,495 and that the level of cross-talk is 30.8% (188,926 of 611,495). In contrast, the level of cross-talk is only 3.1% using the reference dataset (6,033 of 194,582 non-redundant sRNAs, Additional file 2: Table S1). Likewise, cross-talk also increased with the *Gypsy* families using MASiVEdb, for example from 0.2 to 5.3% between *Ji* and *Huck*, and from 0.2 to 10% between *Opie* and *Cinful-zeon*.

### Normalization: complexities regarding the use of M_sRNAs

#### Exclusion of M_sRNAs in TE studies

The handling of sRNAs with multiple mapping locations is an issue that has long troubled scientists. Often, in an effort to avoid methodological complications, M_sRNAs are excluded from analyses [29, 30, 39–41]. However, even though U_sRNAs correlate more consistently with TE silencing than M_sRNAs [24], a significant proportion of RNA-directed DNA methylation (RdDM) is thought to be mediated by M_sRNAs [38]. Moreover, our data in Fig. 2b suggest that there may not be enough U_sRNAs (at least for genome-wide TE annotations) to make meaningful inferences about TEs in hosts with large genomes.

To examine potential U_sRNA differences among plant species with varying genome sizes, we calculated the median density of 24 nt U_sRNAs per nucleotide of maize TEs (for all libraries and families combined) and compared it to those of *Arabidopsis thaliana* and *lyrata* TEs previously reported by Hollister et al. [24]. While the median densities were only twofold different between *thaliana* and *lyrata* (0.11 vs. 0.06), these two species had a 69-fold and 37-fold difference with maize respectively (0.0016 24 nt U_sRNAs per nucleotide of maize TEs). Comparative data were not available for 21–22 nt U_sRNAs from [24], but given that only 3,522 21-22 nt U_sRNAs from all libraries mapped to the 15,532 full-length elements of the *Copia* and *Gypsy* datasets combined, it is clear that most elements did not map U_sRNAs in maize.

#### Normalization of M_sRNAs across genomic regions and between datasets

Besides excluding M_sRNAs from analyses or sometimes even allocating them randomly to single loci [49–51], the most common approaches for handling M_sRNAs is either to count all mapping locations so that each location has a value of 1.0, or to weight for multiple mapping so that each location is assigned a value of 1/*x*, where *x* is the total number of locations for a given M_sRNA. This normalization can be applied to both ‘sRNA species’ and ‘sRNA expression’. Nonetheless, it is unclear if and how these normalization strategies affect downstream research. One parameter that may provide valuable insights is the number of mapping locations for M_sRNAs that target various parts of a genome or different reference datasets. The reasoning is that the smaller the *x*, the weaker the differences between strategies will be and *vice versa*. We therefore compared the mapping locations of M_sRNAs that target our *Copia* and *Gypsy* families i) across the genome, ii) within their annotated full-length populations, and iii) across the TE exemplar database (Fig. 1), so as to keep in line with the various strategies of previous studies.

Focusing first on the entire maize genome, we find that M_sRNAs have an exceptionally high number of mapping locations. For example, the median number of locations for all families combined was up to 513 among the three libraries, while the average often exceeded 1,500 (Table 1). Second, there was a marked decrease in the number of locations within the annotated full-length populations (Table 1). We found that, on average, only a fifth of the genomic locations correspond to full-length elements, indicating that most M_sRNAs map to other types of sequences related to the six families, presumably unidentified full-length elements, degraded copies or solo LTRs. Third, the decrease was even more dramatic within the TE exemplar dataset, where the M_sRNAs of the six families only had three to five mapping locations each (Table 1).

**Table 1:** Number of locations for M_sRNAs that mapped to different parts of the maize genome

library	sRNA length	# of locations for sRNAs of the six families^a^			# of genomic loci for exon sRNAs^a^	# of genomic loci for other sRNAs^a^
		genome	annotated TE population	TE exemplars		
leaf	21	283 – 1397	66 – 298	3 – 5	4 – 12	5 – 37
	22	262 – 1261	70 – 284	3 – 5	4 – 11	5 – 42
	24	82 – 613	11 – 121	3 – 4	4 – 12	4 – 21
	all	127 – 854	18 – 179	3 – 4	4 – 11	4 – 26
tassel	21	425 – 2033	114 – 419	3 – 5	4 – 18	6 – 57
	22	380 – 1615	118 – 369	3 – 5	4 – 15	7 – 60
	24	199 – 1017	26 – 194	3 – 4	5 – 17	4 – 25
	all	277 – 1353	60 – 281	3 – 5	5 – 17	4 – 34
ear	21	513 – 2130	86 – 411	4 – 5	4 – 14	6 – 55
	22	454 – 1748	83 – 359	4 – 5	4 – 15	7 – 56
	24	147 – 897	19 – 170	3 – 5	4 – 17	5 – 26
	all	219 – 1231	31 – 241	3 – 5	4 – 16	5 – 32

^a^The median (left) and average (right) number of mapping locations are shown for each category

The above findings were derived from the most abundant TE families in maize and hence represent the most repetitive parts of a large genome. To contrast them with lower copy regions, we calculated the genomic locations of two additional sets of M_sRNAs: M_sRNAs that mapped to exons of the maize Filtered Gene Set and all other M_sRNAs that did not map to either exons or the six TE families (Fig. 1). We assume that a substantial proportion of the last category corresponds to less abundant TE families. Our analysis showed that the mapping locations of both categories did not exceed a handful of sites (Table 1); nonetheless, the average number of locations of the ‘other’ M_sRNAs was three-fold higher than the exon-mapping M_sRNAs, implying that a large proportion of the former type may indeed map to low copy TEs.

#### Impact of normalization on data inference

**Fig. 4:**
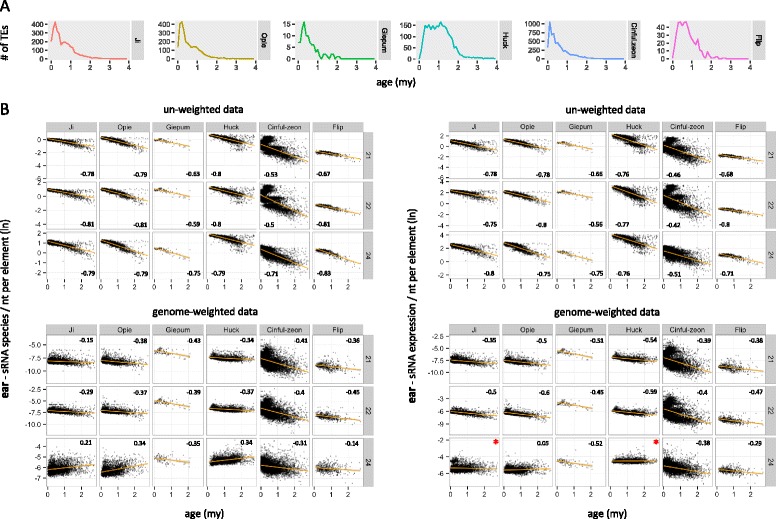
Relationship between TE age and sRNA mapping using un-weighted and genome-weighted approaches. **a** Age distribution in million years (my) of TE families. **b** Mapping of sRNA species (*left panels*) or expression (*right panels*) from ear tissue was calculated per nucleotide of full-length elements for each family. Age is cutoff at 3my to allow sufficient visualization of the *x*-axis. The Spearman *r* coefficient is shown for each plot, calculated for all elements and not only for those <3my. *P* values were <0.01, except those indicated by an asterisk

To gain further insights into how sRNA metrics can change as a function of methodology, we compared the two extremes of a theoretical ‘normalization spectrum’, i.e. un-weighted vs. genome-weighted sRNA data, in their relationship with a classic TE variable, the TE insertion age. The age of each element was first calculated based on the sequence divergence of the LTR pair and profiled at the family level (Fig. 4a). Use of un-weighted data generated strong negative correlations between age and both sRNA species and sRNA expression for all combinations of tissue, family and sRNA length (average Spearman *r* = −0.67, *P* < 10^−20^; Fig. 4b, Additional file 1: Figure S3). Critically, use of genome-weighted data retained this pattern only for 21–22 nt sRNAs (average Spearman *r* = −0.35, *P* < 10^−20^ in most cases), while for 24 nt sRNAs there was discordance both between sRNA metrics and among families. We detected a positive correlation for *Ji*, *Opie* and *Huck* using sRNA species, which was often reversed or not statistically supported using sRNA expression (Fig. 4b, Additional file 1: Figure S3). In contrast, there was a negative correlation for *Cinful-zeon*, *Flip* and *Giepum* across most tissues and for both sRNA metrics.

#### U_sRNA-guided mapping of M_sRNAs

An alternative approach for mapping M_sRNAs assigns reads to single loci using as guide the local densities of U_sRNAs [18]. This method, which is at the core of the ShortStack tool [12], aims to find the true generating locus of each read. Historically, this concept was initially tested with mRNA data where it significantly improved placement of M_mRNAs [52]. For sRNAs, recent analysis of simulated libraries by [18] showed that the U_sRNA-guided mode outperforms other methodologies in selecting the correct locus from which an M_sRNA may have originated.

**Fig. 5:**
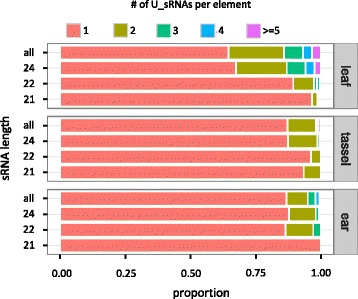
Proportion of the number of U_sRNA species that mapped per TE

However, our data suggest that two properties of TEs may pose a real challenge to this process. First, there is a very small number of U_sRNAs that align to our TEs. For example, only 2,166 of 147,034 sRNA species of the ear library that collectively mapped to *Copia* and *Gypsy* elements are U_sRNAs (Fig. 2b, Additional file 2: Table S2); furthermore, the vast majority of these U_sRNAs mapped to different TEs (Fig. 5). As a result, and given that the length of our TEs ranges between 7–15 kb and that ShortStack examines 250 nt windows [18], it is expected that most windows will not have a U_sRNA score and hence vast amounts of M_sRNAs will be discarded. The second issue concerns the numerous genomic locations for M_sRNAs mapping to TEs (Table 1). These are far above the 50-target cutoff that [18] suggest leads to a high rate of misplacement. Finally, ShortStack can also guide M_sRNA allocation by calculating the densities of both U_sRNAs and weighted M_sRNAs; however, this option did not perform as well as the U_sRNA-only option at the genome level in Arabidopsis, rice and maize [18] and, hence, it is likely that its performance will be further compromised in TE-focused analyses.

### sRNA metrics: unexpected differences between sRNA species and sRNA expression

**Fig. 6:**
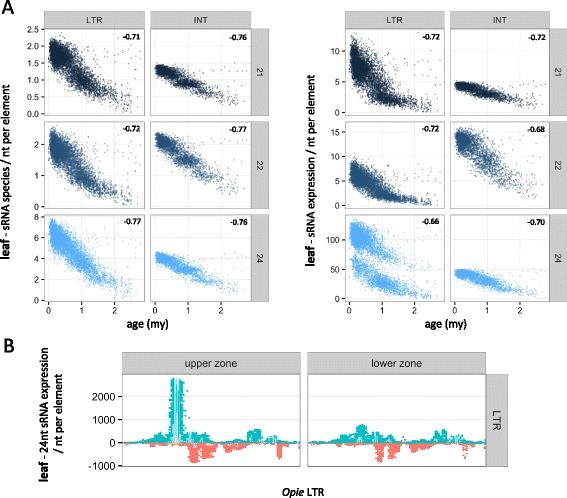
*Opie* population split based on sRNA expression data from leaf tissue. **a** Relationship between TE age and number of sRNA species (*left*) or expression (*right*) calculated per nucleotide of the *Opie* LTRs and INT domain. Age is cutoff at 3my to allow sufficient visualization of the *x*-axis. The Spearman *r* coefficient is shown for each plot, calculated for all elements and not only for those <3my. **b** Mapping patterns (calculated as in Fig. 3a) of 24 nt expression data along the LTRs of the two distinct *Opie* subpopulations. sRNA data in A and B were not weighted by their number of genomic loci

So far, our analysis has indicated that sRNA species and sRNA expression generally produce similar results. However, this is not always true. When we examined the relationship between sRNAs and age separately for the LTRs and the INT domain of TEs using un-weighted data, we observed that the plots of the *Opie* family were markedly different in one case. The expression levels of 24 nt sRNAs from leaf on the LTRs split the *Opie* elements in two distinct groups, whereby the ‘upper zone’ was mapped by approximately twice as many reads compared to the ‘lower zone’ (Fig. 6a). Species of 24 nt sRNAs did not generate the same pattern, nor did other combinations of sRNA lengths and metrics in *Opie* (Fig. 6a), or in other families or tissues (not shown).

Closer investigation revealed that this ‘zoning’ was triggered by sRNAs that mapped to a narrow region on the sense strand of the LTRs (Fig. 6b). This region was mapped by ~115x more reads in the elements of the upper zone compared to those of the lower zone (median coverage of 1,610 and 14 reads/nt respectively), while there was only a three-fold difference (6.1 vs. 2.1 reads/nt) along the rest of the LTR. This implied that highly expressed sRNA species mapping to this region of the elements of the upper zone caused the *Opie* split. We retrieved 836 24 nt sRNA species from all *Opie* elements and, surprisingly, only one appeared to be responsible for the zoning. This sRNA combined very high expression (1,976 reads) and number of mapped LTRs (3,228), ranking 1^st^ and 7^th^ respectively among the 836 sRNAs. In contrast, most other sRNAs of the same region had expression levels of <10 reads.

## Discussion

In this work, we attempted to address the complex issue of mapping and analyzing sRNAs in the context of TEs, which comprise the majority of animal and, especially, plant genomes.

### Reference datasets

Our first objective was to compare mapping characteristics of TE exemplars vs. annotated TE populations, using the large and TE-rich maize genome as a case study. TE exemplars have been widely popular thus far, because of the absence of sufficient sequence information for many species or, perhaps, because research would not truly benefit from the burdensome analysis of annotated TE populations. However, our results indicate that the usage of exemplars comes with several limitations. We showed that a substantial fraction of sRNA information is lost when using exemplars (Fig. 2a, Additional file 2: Table S1). In addition, U_sRNAs are falsely overrepresented in exemplar datasets (Fig. 2b, Additional file 2: Table S2) and hence their use over M_sRNAs (e.g., [29, 30]) should be carefully considered. Finally, and perhaps most importantly, exemplars may entirely omit mapping to specific regions of TEs – most likely, those regions that evolve rapidly within a TE family (Fig. 3).

Yet, our analysis implies that a fraction of annotated TE populations may contain foreign TE fragments, or TE ‘contamination’. It is likely that some types of epigenetic analyses, for example (and as shown earlier) research on sRNA ‘cross-talk’ between TE families implicated in spreading silencing through homology-based defense mechanisms [36, 37], might be negatively affected by this type of ‘contamination’. Hence, it is advisable that careful filtering for foreign DNA is considered prior to mapping sRNA data.

### Normalization

Our next objective was to examine if and how different strategies for treating M_sRNAs might affect biological inference. First, we showed that the inclusion of M_sRNA reads is necessary in TE studies, because U_sRNAs alone may convey little information at the genome level for maize and other species that do not have unusually small genomes.

We then explored the extent of multiple mapping for sRNAs across different genomic regions or datasets in maize. We found that there can be up to a hundred-fold variation in the number of locations for M_sRNAs on maize TEs depending on the reference dataset (Table 1), especially for high-copy TEs. Furthermore, it is likely that this holds true for the majority of plants, as most species have genomes larger than maize with concomitant TE content [23].

Next, we analyzed the relationship between sRNA mapping and TE age using un-weighted vs. genome-weighted data. Among the few studies that have investigated this relationship, most have shown that older TEs map lower levels of sRNAs than younger TEs [24, 25, 53] – a finding which agrees with the expectation that old TEs are deeply silenced and maintained in this state independently of sRNAs [36, 54]. However, one recent study found the opposite trend [55], making this a controversial topic. We found clear evidence for an inconsistent relationship between 24 nt sRNAs and age as a function of methodology (Fig. 4b, Additional File 1: Figure S3), suggesting that the choice of treatment of HTS data can indeed affect biological inference. In contrast, the conclusions based on the other sRNA lengths were unchanged, always generating a negative correlation between sRNA mapping and age (Fig. 4b, Additional File 1: Figure S3). At first sight, this consistency may appear counterintuitive because (as mentioned earlier) weighting-by-location is expected to have a stronger impact on high-copy than low-copy sequences. Yet, 21–22 nt sRNA profiles did not change as a function of age within each family, whereby the numerous young and highly similar elements were mapped by more sRNAs than their few, old and divergent relatives in both normalization approaches. We argue that these findings offer strong support for decreasing levels of 21–22 nt sRNAs as TEs become older, while further research is required to resolve the relationship between 24 nt sRNAs and TE age.

We lastly investigated whether approaches that assign M_sRNAs to single loci based on U_sRNAs density are applicable to TE studies. We concluded that, although promising, this might not be the case yet. Nonetheless, our analysis prompts another point that is well worth discussing. We believe that a distinction is missing – and should be made – between approaches for finding sRNA-generating loci vs. sRNA-targeting loci. For example, ShortStack appears to work beautifully for allocating M_sRNAs to their single locus of origin, which may be valuable in miRNA studies or when organisms have small genomes as in the case of *Arabidopsis thaliana* [18]. However, studies that investigate sRNA targeting patterns may benefit more by methods that allow multiple mapping (weighted or un-weighted). This may be important for TEs, where it is possible that a given sRNA mediates silencing of more than one locus. Although not empirically proven yet, this conjecture is supported by evidence for the importance of M_sRNAs in RdDM [38], the homology-based *trans* silencing pathway among TEs [37], and the cytoplasmic step of Argonaute loading that dissociates sRNAs from their generating loci [56].

#### Normalization and inference for RNA-seq HTS data

**Fig. 7:**
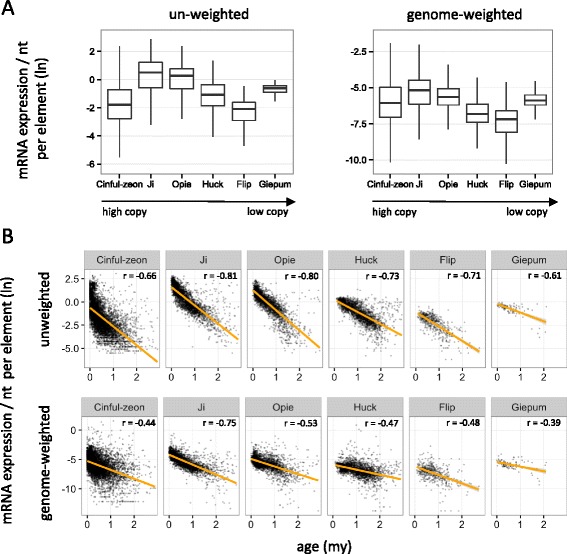
Comparison of un-weighted and genome-weighted mRNA expression data mapping to TEs. **a** Family expression patterns. **b** Relationship between TE age and mRNA mapping. Age is cutoff at 3 million years (my) to allow sufficient visualization of the x-axis. The Spearman r coefficient is shown for each plot, calculated for all elements and not only for those <3my. *P* values were <0.01 in all cases. Library SRR531869 was used for A and B, because mapping patterns of the three replicate libraries to individual elements of the six families were highly correlated (Additional file 1: Figure S4)

We expanded our analysis by investigating mRNA expression data – the most common type of RNA-seq HTS data. Although the proportion of multiply mapping reads against the genome in these libraries is only ~10% and substantially lower to the 40–90% of sRNA libraries [18], it is likely that a much higher proportion of TE-mapping reads will be categorized as M_mRNAs. As a result, similar methodological complications to TE epigenetic studies may apply to studies examining TE expression [57]. We therefore retrieved mRNA HTS data from three biological leaf replicates and examined (as we did with sRNAs) i) their general mapping characteristics, ii) the expression patterns of TE families, and iii) the relationship between expression and TE age. First, we found that the vast majority of TE-mapping reads were indeed M_mRNAs (~90%, Additional file 2: Table S3); the median number of locations for these M_mRNAs across the genome or within the annotated full-length elements (Additional file 2: Table S3) was approximately two-fold lower to those of the TE-mapping M_sRNAs (Table 1). Second, the use of either un-weighted or genome-weighted data generated the same relative expression levels among families despite their widely different sizes (Fig. 7a). Finally, both normalization approaches produced strong negative correlations between mRNA expression and age for all possible combinations (average Spearman *r* = −0.61, *P* < 10^−20^; Fig. 7b). These findings suggest that, at least for the specific inquiries, the methodological treatment of RNA-seq HTS data does not change biological inference.

### sRNA metrics

Our final objective was to test for differences derived from using the metrics of sRNA species or sRNA expression. We did identify an unexpected inconsistency in relation to a narrow region in the *Opie* LTRs, whereby the very high expression of a single sRNA species was able to split the LTRs into two distinct zones with and without the target sequence (Fig. 6). Albeit very intriguing, the fact that only one sRNA generated this spectacular pattern raises several methodological concerns. First, it is likely that such very high expression levels may be the outcome of biases during library construction [15]. Second, our data imply that the use of sRNA species is more robust than sRNA expression, because it appears to be less sensitive to errors that can occur, e.g., during PCR amplification. Finally, and perhaps most importantly, these findings denote the need for the confirmation of such observations. This can be achieved by cross-examining results from different normalization approaches. However, given the inconsistencies of normalization approaches as discussed previously, the most appropriate way is the inclusion in the experimental design of technical and/or biological replicates. In previous years, the lack of sRNA replicates could be attributed to the high costs of sequencing. These costs are now much lower and, hence, replicates should be typically included in epigenetic studies to help identify aberrancies.

## Conclusions

The epigenetic interactions between TEs and host defense mechanisms have been the focus of intensive research for several years now. These studies often include the mapping and analysis of HTS sRNA (and mRNA) data to TE sequences. However, the complications of mapping short reads to repeated and difficult-to-annotate DNA sequences have not been given enough attention, allowing scientists to follow various, often conceptually opposite, methodologies in their work. Our goal here was to fill this gap. Even though we did not empirically test or provide solutions for some of these issues (e.g. to resolve the 24 nt sRNA vs. TE age relationship or to find the generating locus of TE sRNAs), we aim to make our peers at least aware of these complications and help guide future studies. Towards this aim, we can provide the following take-home messages:

1. TE exemplars should be – at best – cautiously used, and replaced with annotated TE populations (additionally curated, if needed) whenever possible.
2. The inclusion of multiply mapping sRNA and mRNA reads is necessary, in TE studies, especially in large and complex genomes.
3. Weighted and un-weighted mapping strategies should be used in parallel to help validate biological inferences.
4. Fully, or even partially, sequenced genomes should be preferred over exemplars for weighting-by-location of multiply mapping reads.
5. sRNA expression – a crucial metric for differential expression analysis studies – is prone to errors during HTS library preparation, and therefore, the inclusion of replicates in sRNA studies should now be standard.

## Additional files

Additional file 1: Figure S1. Gypsy TEs length analysis. **Figure S2.** Total number of sRNA species that mapped to different datasets of the three *Copia* families. **Figure S3.** Relationship between TE age and sRNA mapping for leaf, tassel and ear tissues. **Figure S4.** Mapping patterns of three leaf replicate mRNA libraries to individual elements using un-weighted data. (PDF 5159 kb)
Additional file 2: Table S1. Number of sRNA species mapping to different TE reference datasets. **Table S2.** Mapping of U_sRNA and M_sRNA species to exemplars and annotated TE populations for all families combined. **Table S3.** Size and mapping characteristics of mRNA libraries. (DOCX 102 kb)

## Abbreviations

env: Envelope
HTS: High-throughput sequencing
INT domain: Internal domain
LTR: Long terminal repeat
M_sRNA: Multiply mapped sRNA
miRNA: micro RNA
sRNA: small RNA
TE: Transposable element
U_sRNA: Uniquely mapped sRNA
