# Supplementary material for: Considerations and complications of mapping small RNA high-throughput data to transposable elements
Source: Mob DNA. 2017 Feb 15;8:3. doi: 10.1186/s13100-017-0086-z (PMC5311732; doi:10.1186/s13100-017-0086-z)
Supplement: Additional file 1: Figure S1. — Gypsy TEs length analysis. Figure S2. Total number of sRNA species that mapped to different datasets of the three Copia families. Figure S3. Relationship between TE age and sRNA mapping for leaf, tassel and ear tissues. Figure S4. Mapping patterns of three leaf replicate mRNA libraries to individual elements using un-weighted data. (PDF 5159 kb) [file 13100_2017_86_MOESM1_ESM.pdf]

Figure S1

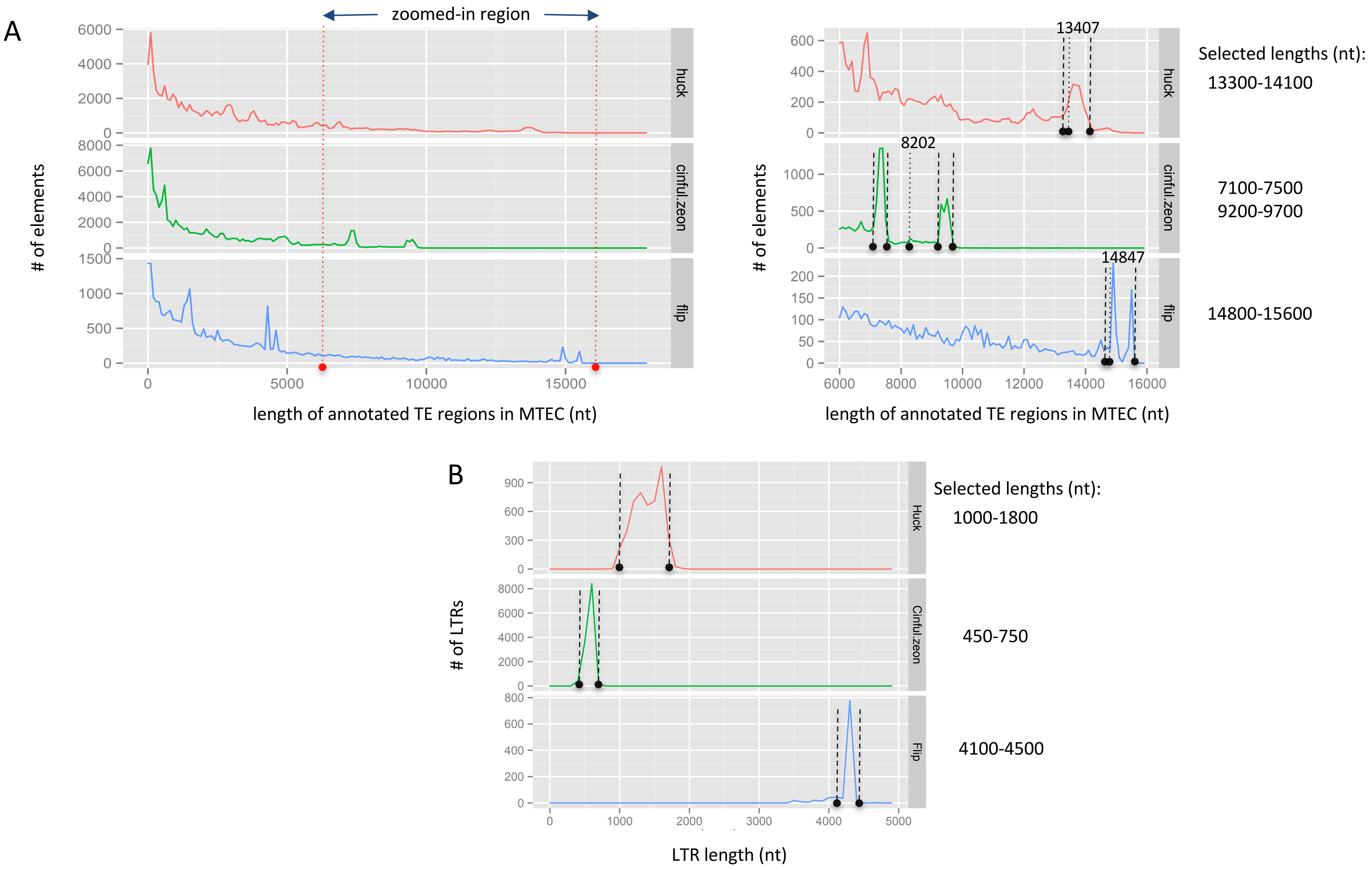

Figure S1. Gypsy TEs length analysis. (**A**) Frequency distribution of the lengths of annotated TE region according to the maze TE consortium. (**B**) Frequency distribution of LTR lengths. Thick dotted black lines indicate the full-length and LTR ranges used for each family, while the thin dotted black lines and number at the top of each plot in **A** show the Baucom et al. (2009) average.

Figure S2

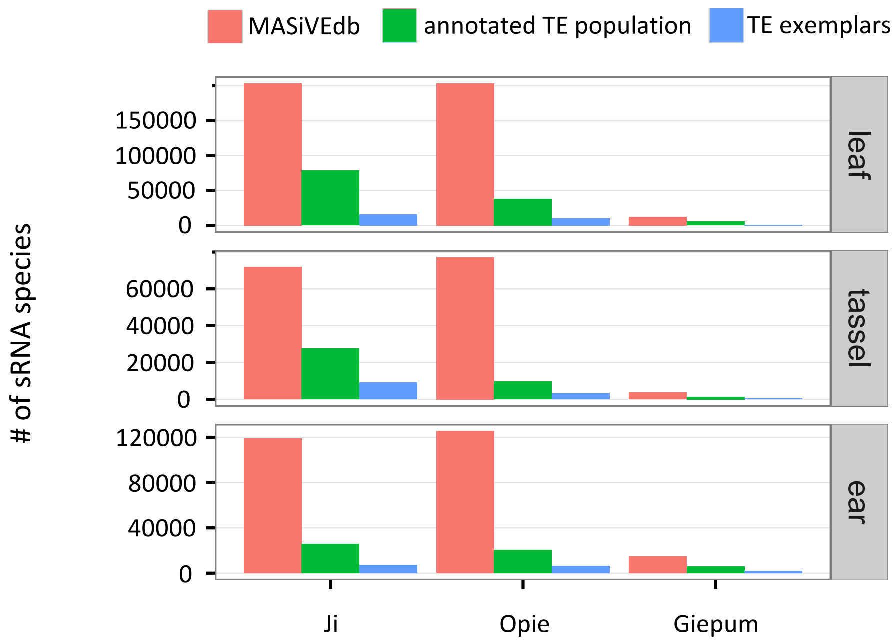

Figure S2. Total number of sRNA species that mapped to different datasets of the three *Copia* families .

Figure S3

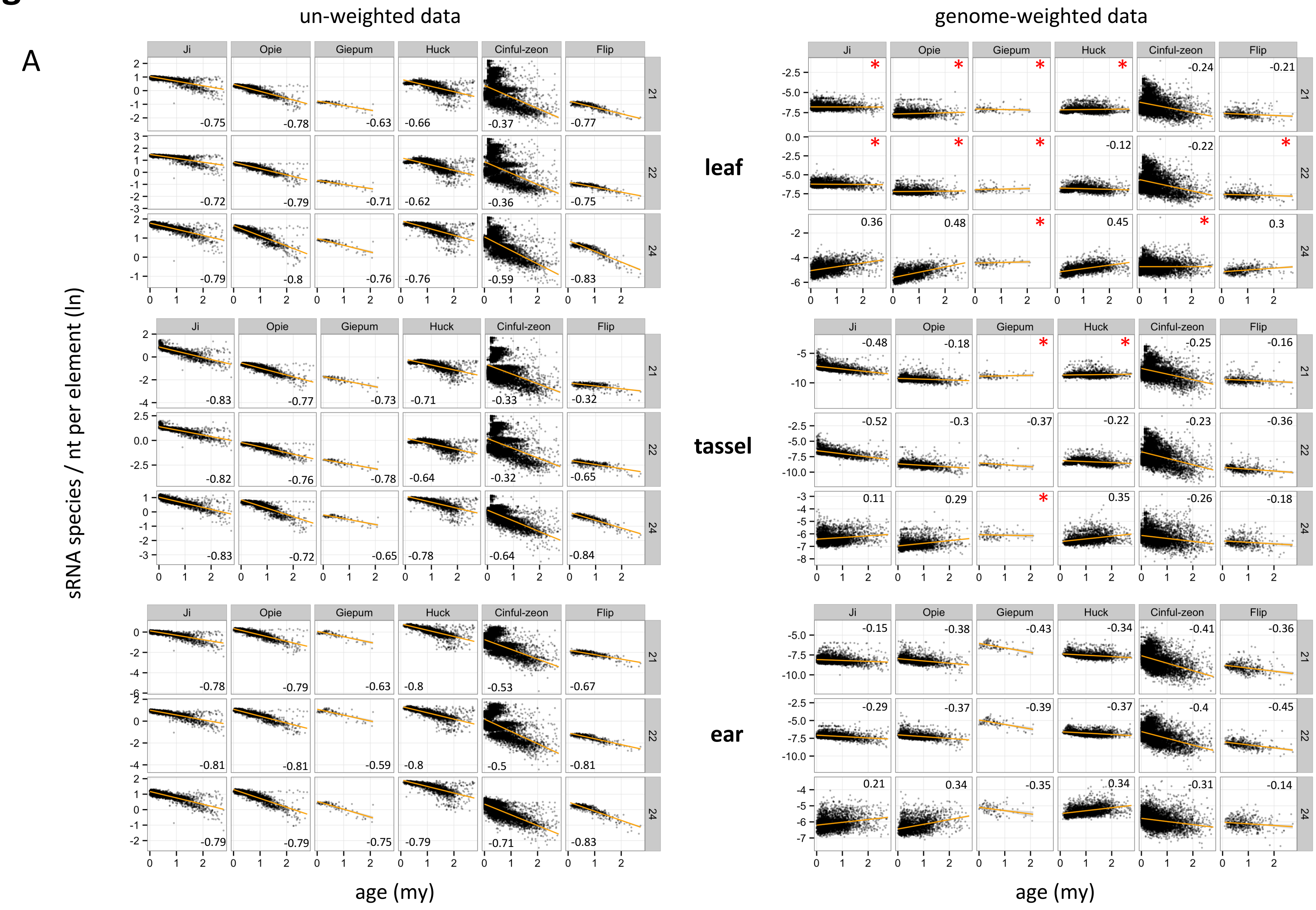

Figure S3. Relationship between TE age and sRNA mapping for leaf, tassel and ear tissues. The number of sRNA species (**A**) or expression (**B**) was calculated per nucleotide of full-length elements for each family, using un-weighted (left panels) and genome-weighted (right panels) approaches. Age is cutoff at 3 million years (my) to allow sufficient visualization of the *x*-axis. The Spearman *r* coefficient is shown for each plot, calculated for all elements and not only for those <3my. *P* values were <0.01, except those indicated by an asterisk.

Figure S3 (cont.)

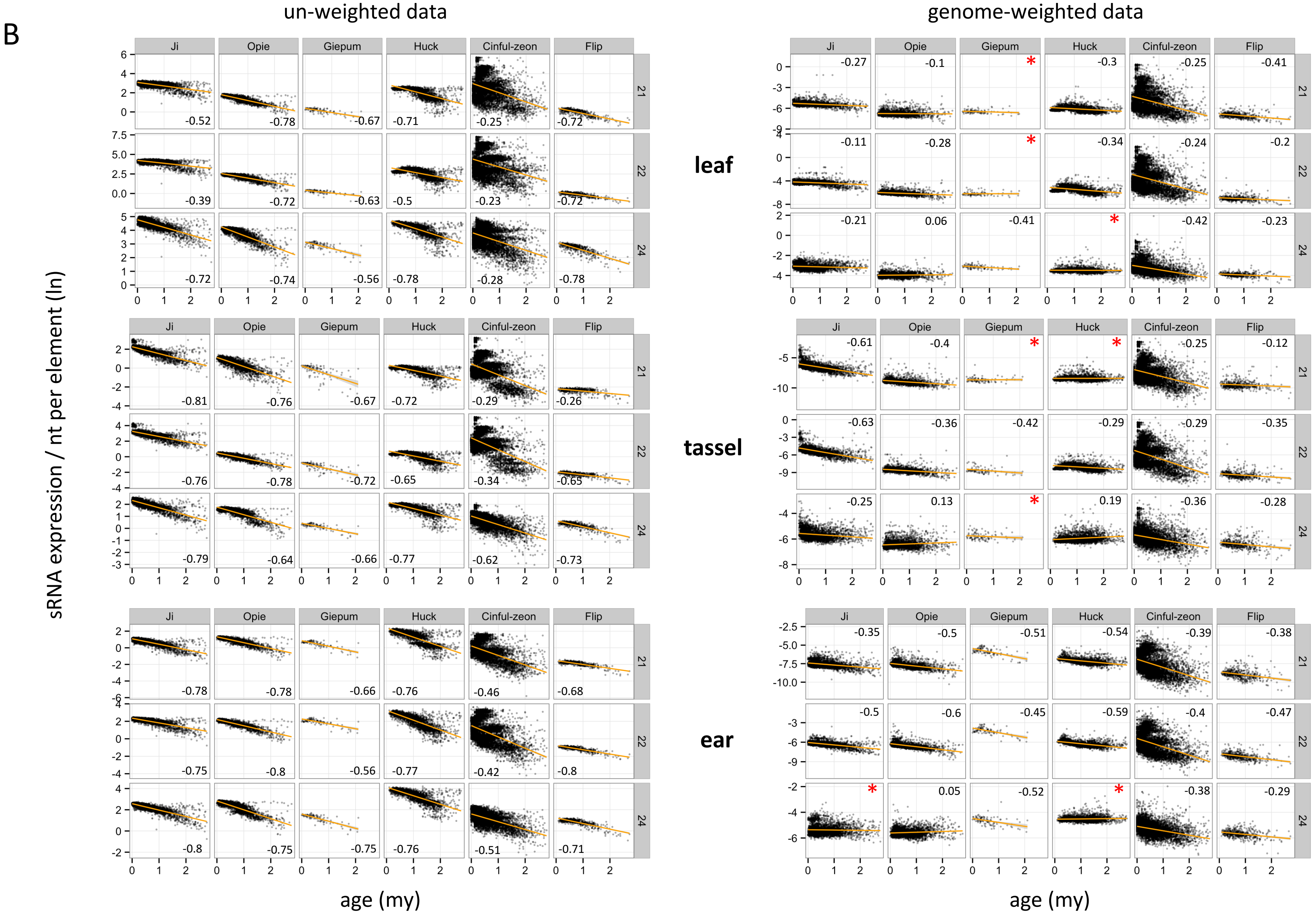

Figure S4

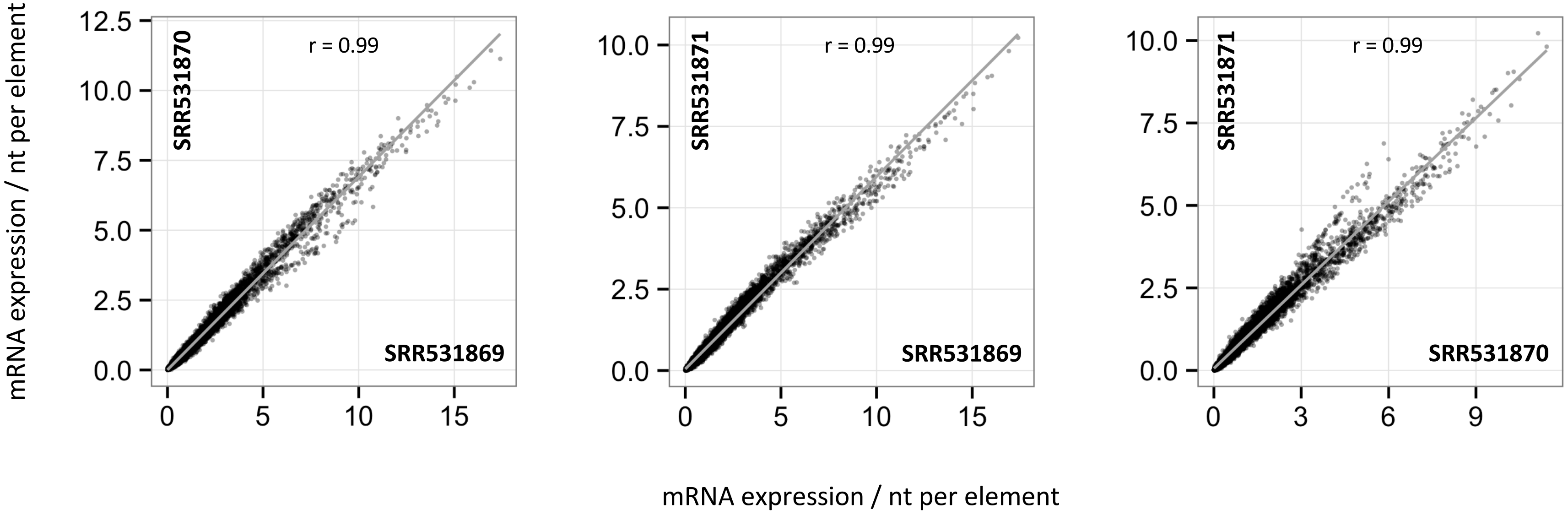

Figure S4. Mapping patterns of three leaf replicate mRNA libraries to individual elements using un-weighted data. The Spearman *r* coefficient is shown for each plot. *P* values were <0.01 in all cases.
