# Supplementary material for: Considerations and complications of mapping small RNA high-throughput data to transposable elements
Source: Mob DNA. 2017 Feb 15;8:3. doi: 10.1186/s13100-017-0086-z (PMC5311732; doi:10.1186/s13100-017-0086-z)
Supplement: Additional file 2: Table S1. — Number of sRNA species mapping to different TE reference datasets. Table S2. Mapping of U_sRNA and M_sRNA species to exemplars and annotated TE populations for all families combined. Table S3. Size and mapping characteristics of mRNA libraries. (DOCX 102 kb) [file 13100_2017_86_MOESM2_ESM.docx]

**Table S1.** Number of sRNA species mapping to different TE reference datasets

| **dataset** | **library** | *Ji* | *Opie* | *Giepum* | ***Copia*^b^** | *Huck* | *Cinful-zeon* | *Flip* | ***Gypsy*^b^** | **all^c^** |
| --- | --- | --- | --- | --- | --- | --- | --- | --- | --- | --- |
| **TE exemplars** | **leaf** | 15,955 | 10,040 | 1,282 | **27,126** | 21,113 | 37,607 | 4,657 | **63,377** | **90,503** |
|  | **tassel** | 9,176 | 3,239 | 333 | **12,653** | 7,342 | 17,085 | 1,441 | **25,868** | **38,521** |
|  | **ear** | 7,160 | 6,406 | 2,125 | **15,620** | 20,915 | 12,955 | 2,674 | **36,544** | **52,164** |
| **annotated TE population** | **leaf** | 78,714 | 38,187 | 5,645 | **119,221** | 74,760 | 101,735 | 16,883 | **192,878** | **310,548** |
|  | **tassel** | 27,694 | 9,657 | 1,395 | **37,286** | 19,062 | 33,785 | 3,936 | **56,567** | **93,173** |
|  | **ear** | 25,989 | 20,374 | 5,929 | **50,082** | 58,525 | 31,654 | 7,058 | **96,952** | **147,034** |
| **complete *Copia* population (MASiVEdb^a^)** | **leaf** | 203,309 | 203,385 | 12,273 | **318,151** | same number of sRNA  species as above | | | | **457,081** |
|  | **tassel** | 71,847 | 77,180 | 3,719 | **110,455** |  |  |  |  | **145,413** |
|  | **ear** | 119,208 | 125,492 | 14,749 | **198,230** |  |  |  |  | **263,946** |

^a^ The complete annotated populations for the *Ji*, *Opie* and *Giepum* families were retrieved from MASiVEdb ([bat.infspire.org/databases/masivedb/](http://bat.infspire.org/databases/masivedb/)). See Figure 1 for the number of full-length elements of each dataset.

^b^ These two columns present the non-redundant sums of the previous three, i.e. after taking into account that a (low) number of sRNA species map to multiple TE sets or in other words participate in sRNA ‘cross-talk’ (as described in the text) – counting those sRNA species only once results in numbers in these two columns lower than the actual sums of the preceding columns.

^c^ As in the previous footnote, this column presents the non-redundant sums of all six TE families.

**Table S2.** Mapping of U_sRNA and M_sRNA species to exemplars and annotated TE populations for all families combined

| sRNA type | library | TE exemplars | annotated TE population | genome |
| --- | --- | --- | --- | --- |
| U_sRNAs | leaf | 41,718 | 10,828 | 2,703,286 |
|  | tassel | 16,643 | 1,650 | 749,733 |
|  | ear | 19,182 | 2,166 | 613,801 |
| M_sRNAs | leaf | 48,785 | 299,720 | 1,959,315 |
|  | tassel | 21,878 | 91,523 | 605,229 |
|  | ear | 32,982 | 144,868 | 788,452 |
| % of U_sRNAs | leaf | 46.1 | 3.5 | 58.0 |
|  | tassel | 43.2 | 1.8 | 55.3 |
|  | ear | 36.8 | 1.5 | 43.8 |

**Table S3**. Size and mapping characteristics of mRNA libraries

| mRNA libraries | U_mRNAs^a^ | M_mRNAs^a^ | SRR531869 | M_mRNA  category^b^ | # of genomic loci | | # of loci within TE population | |
| --- | --- | --- | --- | --- | --- | --- | --- | --- |
|  |  |  |  |  | median | avg. | median | avg. |
| SRR531869 | 21,969,195 (4,331) | 2,700,932 (30,165) |  | annotated TE population | 137 | 376 | 29 | 78 |
| SRR531870 | 14,459,552 (2,630) | 1,655,471 (20,073) |  | exons | 2 | 4 |  | |
| SRR531871 | 15,067,304 (1,969) | 1,905,097 (17,569) |  | other | 15 | 133 |  |  |

^a^Numbers in parentheses indicate the mRNA reads that mapped to our *Copia* and *Gypsy* elements.

^b^Each M_mRNA read of library SRR531869 was grouped according to whether it mapped to our reference TE dataset, to exons, or to neither of these regions.
